# Building a world-class emergency medicine system: Qatar’s strategic evolution and outcomes

**DOI:** 10.5339/qmj.2026.37

**Published:** 2026-06-09

**Authors:** Shahzad Anjum, Alreem Alkuwari, Khalid Bashir

**Affiliations:** 1 Department of Emergency Medicine, Hamad Medical Corporation, Doha, Qatar; 2 Clinical Department, College of Medicine, QU Health, Qatar University, Doha, Qatar

**Keywords:** Emergency medicine, Qatar, healthcare systems, medical education, graduate medical education, healthcare reform

## Abstract

This narrative review examines the evolution, organization, and performance of emergency medicine (EM) services in Qatar within a nationally coordinated healthcare system. Qatar operates a highly centralized public healthcare model serving approximately 2.9 million residents through a single national health region, which includes 31 primary healthcare centers and multiple secondary and tertiary hospitals, encompassing both teaching and non-teaching institutions. Emergency departments manage a wide spectrum of acute presentations, most commonly trauma, cardiovascular emergencies, respiratory illnesses, and undifferentiated medical conditions. The review explores key aspects of workforce development, including structured training pathways, residency programs, and interprofessional education initiatives, placing Qatar’s experience within a broader global context. International literature indicates that challenges related to workforce sustainability, trainee well-being, retention, and standardization of education are widely shared across health systems, highlighting the universal relevance of these issues. Qatar’s nationally coordinated approach to emergency care delivery, residency training, clinical governance, and quality improvement has strengthened workforce capacity, enhanced clinical performance, and improved system resilience. The transformation from a fragmented, generalist-driven service to a cohesive, specialty-led network demonstrates how coordinated planning, structured education, and infrastructure investment can achieve international standards of emergency care. By synthesizing these developments, this review provides insights and transferable lessons for other nations seeking to establish robust, high-quality EM services, illustrating the impact of systematic, evidence-based approaches on both patient care and health system performance.

## 1. INTRODUCTION

Qatar, an independent nation since 1971, is a high-income country on the Arabian Peninsula with a population of over three million.^1^ In line with Qatar National Vision 2030, the nation has made sustained investments in its healthcare system under the oversight of the Ministry of Public Health. Administratively, Qatar is divided into three main regions—Doha, Al Rayyan, and the Northern and Southern municipalities—which vary in population density and socio-economic status. Doha is the most urbanized and affluent region, while the outer municipalities are less densely populated and more rural.

The public healthcare sector in Qatar is led by Hamad Medical Corporation (HMC), one of the largest and most advanced hospital providers in the Middle East. HMC operates a network of over 14 hospitals, including both teaching and non-teaching facilities, delivering secondary and tertiary care across the country. The Corporation provides a wide range of specialized services, including the National Ambulance Service, mental healthcare, home care, and residential services, while also serving as a key institution for postgraduate medical education and training. HMC is committed to ensuring the highest standards of patient safety and clinical effectiveness, supporting Qatar’s strategic vision for a robust, high-quality healthcare system.^2^ Postgraduate medical education at HMC spans multiple specialties, with training programs rigorously evaluated through studies such as Khalil et al.’s examination of urology residents’ perspectives on on-call systems.^3^ Complementing this system, the Primary Health Care Corporation (PHCC) operates 31 health centers across the three regions, providing comprehensive primary care for the community.^4^

Together, HMC and PHCC serve a diverse and rapidly growing population with significant emergency care needs, driven by high rates of trauma from road traffic incidents and an increasing burden of non-communicable diseases such as cardiovascular, respiratory, and metabolic conditions.^2,4^ Emergency departments (EDs) manage a broad spectrum of acute presentations, most commonly trauma, cardiovascular emergencies, respiratory illnesses, gastrointestinal emergencies, and undifferentiated medical conditions, reflecting the population’s evolving health needs. These demographic and socio-economic factors shape ED utilization patterns, influencing both the volume and complexity of acute presentations.

Globally, emergency medicine (EM) was formally recognized as a specialty in the United States in 1968, establishing training and clinical standards that later influenced systems worldwide.^5^ The international expansion of EM has faced recurring challenges related to workforce development, training standardization, and health system integration.^6^ In the Middle East, EM integration was initially limited by structural and workforce constraints. A major regional milestone was the establishment of the Arab Board of EM in 2000, which introduced standardized training frameworks. Qatar recognized EM as a specialty in the same year—a strategic decision aligned with broader national healthcare modernization.^7^

In parallel, Qatar’s trauma system advanced from its early development to full verification, culminating in the Hamad Trauma Center achieving international accreditation in 2014. Key milestones included the creation of the Qatar Trauma Registry, major enhancements in pre-hospital care, the development of a Trauma and Critical Care Fellowship, and the launch of the Hamad Injury Prevention Program.^8^ Collectively, these initiatives reflect a comprehensive, evidence-based commitment to improving the quality, efficiency, and outcomes of emergency and trauma care across the entire continuum—from initial patient contact through rehabilitation.

Despite this rapid progress, the literature lacks a comprehensive synthesis of Qatar’s EM evolution. This review addresses that gap by examining key drivers, milestones, and outcomes from the pre-specialization era to Qatar’s current position as a clinical and academic leader. The findings aim to inform EM development in other rapidly modernizing nations. A narrative review was chosen to enable the integration of historical, policy, and system-level developments that are not amenable to systematic synthesis.

## 2. REVIEW METHODOLOGY

This narrative review synthesizes the evolution of EM in Qatar from the pre-specialization era (pre-2000) to 2025. A comprehensive search was conducted using PubMed, Scopus, and Google Scholar with keywords including “Emergency Medicine Qatar,” “Hamad Medical Corporation Emergency Care,” “Qatar Trauma System,” and “Medical Education Qatar,” limited to publications from January 2000 to June 2025. Grey literature was sourced from official Qatari institutional websites (HMC, PHCC, and the Ministry of Public Health). Sources were selected based on their relevance to EM system development, education, infrastructure, and national outcomes.

The review was organized around four thematic domains:

1. **Education and Training**: the evolution of residency programs and academic milestones.
2. **Infrastructure and Clinical Services**: facility expansion, pre-hospital care, and technological advancement.
3. **Academic and Subspecialty Development**: board certification and fellowship programs.
4. **System Outcomes and Impact**: workforce trends, retention, and national health system performance.

## 3. FOUNDATIONAL CHALLENGES: THE PRE-SPECIALIZATION ERA (PRE-2000)

Prior to 2000, emergency care in Qatar was largely delivered through a generalist model, with no formally dedicated EM physicians or standardized clinical protocols in place.^9^ EDs were staffed by a rotating workforce of surgeons, internists, and anesthesiologists, each managing patients according to their own specialty perspective. These challenges were not unique to Qatar; similar workforce shortages, reliance on non-specialist physicians, and fragmented emergency care models were widely reported during the early development of EM in many countries worldwide, particularly in low- and middle-income and rapidly developing health systems.^10^ This approach often resulted in fragmented and inconsistent care, particularly for patients presenting with complex or multi-system emergencies.^2,9^

Several key challenges characterized this pre-specialization era:

- **Dual responsibilities for clinicians**: Physicians were required to manage both emergency duties and their primary specialty responsibilities. This dual role placed considerable strain on service delivery and sometimes delayed critical interventions.
- I**nconsistent, specialty-driven care pathways**: Clinical management was heavily influenced by individual physicians’ specialty training. The absence of a unified, evidence-based triage or resuscitation system led to variability in patient outcomes and inefficiencies in care.
- **Lack of EM-trained leadership**: Without formally trained emergency physicians, there was limited capacity to coordinate services, standardize care, or implement quality improvement initiatives.

Although some infrastructural elements existed—such as gender-segregated patient areas and designated clinical zones, including trauma sections—Qatar’s EDs, like many international EDs at the time, lacked the specialized staffing and structured protocols required for high-quality emergency care.^5,9^

This situation was further exacerbated by rapid demographic growth; Qatar’s population increased by nearly 50% between 1990 and 2000, significantly intensifying the demand for emergency services and highlighting systemic gaps in care delivery.^1^ These pressures underscored the urgent need for formalized EM training programs, structured clinical protocols, and specialized staffing, ultimately setting the stage for the transformation of Qatar’s emergency care system into a competency-driven, specialty-led service.^11^

## 4. THEMATIC ANALYSIS OF EM EVOLUTION

### 4.1. Education and training

The establishment of Qatar’s first EM residency program under the Arab Board in 2000 marked a pivotal milestone in the country’s healthcare development.^11^ The inaugural cohort, consisting of nine physicians, underwent training under a curriculum designed to integrate regional healthcare needs with internationally recognized standards of emergency medical practice. Upon graduating in 2004, these physicians became Qatar’s first formally EM-trained clinicians and assumed roles as early academic leaders, shaping both clinical practice and future educational initiatives within the country.

A significant evolution occurred following Accreditation Council for Graduate Medical Education – International (ACGME-I) accreditation in 2013, which catalyzed a comprehensive transition to a structured, competency-based training model.^11^ Table 1 summarizes the curricular transformation before and after ACGME-I accreditation. The proportion of EM-specific rotations increased significantly, while non-core rotations were eliminated to focus training on essential EM competencies. In addition, intensive care unit (ICU) exposure expanded, providing residents with extensive experience in managing critically ill patients across the medical intensive care unit (MICU), neonatal intensive care unit (NICU), and surgical intensive care unit (SICU).^11^

Educational innovations were implemented to enhance both clinical competence and professional development. These included: simulation-based training, which allowed trainees to practice high-risk, low-frequency scenarios in a controlled environment; milestone-based assessments, ensuring systematic evaluation of skill progression; and structured quality improvement programs, fostering a culture of continuous learning and patient safety.^12–14^

Beyond resident training, the program emphasized faculty development and mentorship, with senior clinicians guiding trainees in clinical reasoning, research, and teaching. Structured mentorship programs paired junior residents with experienced EM physicians to enhance clinical decision-making, professional development, and career planning. Additionally, interprofessional education initiatives were introduced, involving nursing, paramedic, and allied health staff, to strengthen teamwork and collaborative care—critical competencies in high-acuity emergency settings.^15^

Collectively, these initiatives aligned Qatar’s EM residency program with international best practices, producing physicians equipped not only with the knowledge and skills to manage complex emergency cases but also with the academic foundation to contribute to research, teaching, and leadership in the field of EM. This comprehensive approach has helped position Qatar as a regional leader in both EM clinical practice and graduate medical education.

### 4.2. Infrastructure and clinical services

Qatar’s EM system has advanced substantially through sustained investment in both physical infrastructure and clinical technology. A key strategic development was the decentralization of emergency care from Hamad General Hospital (HGH) to a nationwide network, including several satellite hospitals—Al Wakra Hospital (2012), Hazm Mebaireek Hospital (2018), and Aisha Bint Hamad Al-Attiyah Hospital (2022)—as well as specialty centers such as Sidra Medicine. Collectively, these expansions improved geographic access, reduced overcrowding, and enhanced patient flow and management across the healthcare system.^2,16,17^

As part of the system-wide infrastructure supporting emergency care delivery and training, the Qatar Simulation Consortium was formed in 2013 to unify previously independent simulation programs across Qatar’s major healthcare and educational institutions. Its mission is to advance simulation-based education, research, and practice nationwide in alignment with global standards.^18^ The development of dedicated cardiac and stroke emergency units enabled the system to meet international benchmark times for critical interventions such as percutaneous coronary intervention (PCI) and thrombolysis,^19^ reflecting a strong commitment to both quality and outcome-oriented care.

Pre-hospital services also underwent a major transformation. The fleet expanded dramatically from 10 emergency units in 2000 to 167 advanced life support vehicles by 2024,^20^ enhancing the system’s ability to respond rapidly to both routine emergencies and mass-casualty events. This expansion was complemented by the introduction of helicopter emergency medical services (HEMS), which significantly improved coverage in remote or congested areas and ensured timely transport to tertiary care centers.^20^ Collectively, these infrastructure and service developments not only increased capacity and accessibility but also strengthened Qatar’s ability to deliver high-quality, time-sensitive emergency care on a national scale.

### 4.3. Academic and subspecialty advancement

Qatar’s EM system has undergone notable academic growth through the establishment of structured national certification pathways and the development of newly introduced subspecialty fellowship programs. A major milestone was the launch of the Qatari Board of Emergency Medicine in 2020, which provided a nationally governed framework for certification, assessment, and quality assurance. Its rigor was demonstrated when the inaugural cohort successfully passed the Arab Board EM examination in 2023,^13^ underscoring alignments with regional and international standards.

In recent years, Qatar has introduced several new fellowship programs, which, as far as current evidence suggests, are emerging subspecialty programs that represent an innovative model for the region and for EM subspecialization in the Gulf:

- **Emergency Resuscitation and Critical Care (EMRACC)**: Focused on advanced resuscitation, high-acuity procedures, and emerging therapies such as extracorporeal membrane oxygenation (ECMO), thereby expanding national critical care capability.
- **Point-of-Care Ultrasound (POCUS)**: Designed to enhance rapid bedside diagnostics, streamline patient flow, and improve real-time decision-making in acute care settings.
- **Medical Toxicology**: Developed to address region-specific toxicological presentations, including hydrocarbon ingestion, envenomation, and industrial exposures, thereby advancing both clinical care and public health preparedness.

These subspecialty fellowships are supported by the ITQAN Clinical Simulation and Innovation Centre, which provides high-fidelity simulation, procedural training, and interdisciplinary, team-based scenarios that reinforce competency development.^15,18^ Collectively, these initiatives reflect Qatar’s commitment to advancing clinical proficiency, academic excellence, and regional leadership in EM.

### 4.4. System outcomes and impact

The evolution of Qatar’s EM system has yielded tangible improvements in workforce capacity, clinical performance, and system-wide resilience. Since its inception, the residency program has graduated a substantial number of physicians from over 20 countries, reflecting a diverse, internationally trained workforce. A significant proportion have been retained within Qatar’s healthcare system, contributing to national capacity building and continuity of care, while others have migrated abroad. This illustrates both the international recognition of Qatar’s EM training quality and the ongoing challenge of retaining highly trained physicians.

System resilience and operational capacity have been demonstrated during several high-profile national and international events. During the COVID-19 pandemic, Qatar maintained a comparatively low case-fatality rate, in part through the rapid expansion of ICU capacity, the strategic deployment of telehealth services, and efficient coordination across hospital networks, including the transformation of facilities such as Al Wakra Hospital into dedicated COVID-19 centers.^2,21^ These capabilities were further tested and validated during the 2022 FIFA World Cup, when the EM system successfully managed large-scale, high-acuity patient presentations while maintaining international benchmarks for patient safety and response times.^22^

Collectively, these developments underscore the transformation of Qatar’s EM system from a fragmented, generalist-driven service into a mature, specialty-led network capable of supporting both routine emergency care and national-level crisis response.

## 5. CONCLUSION

Qatar’s evolution of EM reflects a rapid and systematic healthcare development process within a unified national system, as outlined in this review. Strategic investments in infrastructure, competency-based residency training, interprofessional education, and subspecialty development have transformed a previously fragmented, generalist-driven emergency care model into a cohesive, specialty-led network. As discussed, these developments have strengthened workforce capacity, enhanced clinical performance, and supported the delivery of emergency care during both routine operations and major system stressors, including the COVID-19 pandemic and the 2022 FIFA World Cup. The integration of education, clinical services, and quality improvement initiatives described throughout this review demonstrates how a centralized approach can support high standards of patient care, training, and service delivery. Collectively, Qatar’s experience provides insights relevant to other healthcare systems seeking to develop structured, sustainable EM services through coordinated planning and evidence-informed implementation.

## ETHICAL APPROVAL

This manuscript does not involve human participants, identifiable data, or intervention-based research; therefore, it does not require ethical approval from an Institutional Review Board.

## DISCLOSURE OF AI USE

During the preparation of this manuscript, the authors used DeepSeek to provide limited assistance with aspects of manuscript review and critical appraisal. All content was independently reviewed, revised, and verified by the authors, who take full responsibility for the final version of the manuscript.

## ACKNOWLEDGEMENT

The authors acknowledge DeepSeek for its limited assistance in reviewing the manuscript. No external funding or additional support was received.

## CONFLICT OF INTEREST STATEMENT

The authors declare no competing interests. The use of DeepSeek was limited and did not influence the study design, data analysis, interpretation of findings, or the decision to submit the manuscript.

## AUTHOR CONTRIBUTIONS

SA conceived the study and provided the data. AA conducted the literature review, contributed to critical appraisal, and assisted with manuscript writing. KB performed the data analysis, conducted the literature review, drafted the initial manuscript, and addressed reviewers’ comments

## Figures and Tables

**Table 1.** Comparison of Residency Rotation Structure Before and After ACGME-I Accreditation

Rotation category	Pre-ACGME-I accreditation, 2000–2004	Post-ACGME-I accreditation, 2013–present
Emergency medicine	28 blocks, including ED, pediatric EM, and trauma	43 blocks, including ED, pediatric EM, and trauma
Subspecialties	20 blocks, including ENT, Urology, Radiology, Psychiatry, MICU, TICU, NICU, Plastic Surgery, and Ophthalmology	5 blocks, including MICU, orthopedics, anaesthesia, TICU, and Gynaecology
Leave	4 blocks	4 blocks
Total blocks	52 blocks	52 blocks

One block represents approximately four weeks. Both curricula comprise 52 blocks, equivalent to a four-year residency programme. ACGME-I, Accreditation Council for Graduate Medical Education International; ED, Emergency department; EM, Emergency medicine; ENT, Ear, nose, and throat; MICU, Medical intensive care unit; NICU, Neonatal intensive care unit; TICU, Trauma intensive care unit.
